# Validation of the Korean Movement Culture Perception (KMCP) Scale Using the Rasch Model: Evidence from Korean and Chinese Populations

**DOI:** 10.12688/f1000research.171305.1

**Published:** 2026-01-05

**Authors:** Hye-Ryeon Kim, Eunhye Jo, Xuanshang Jin, So-Eun Lee, Chang-Hwan Choi

**Affiliations:** 1 Institute of Physical Education and Science, Korea National Sport University, Songpa-gu, Seoul, South Korea; 2 Physical Education, Korea National University of Education, Cheongju, South Korea; 3 School of Kinesiology and Health Promotion, Dalian University of Technology, China, China; 4 Wesley College of Convergence and Creative Studies, Hyupsung University, gyeonggi-do, South Korea; 5 physical education, Kangwon National University College of Natural Sciences, Chuncheon-si, Gangwon-do, South Korea

**Keywords:** movement culture; embodied cultural practices; Korean Wave (Hallyu); Rasch model; perception scale; K-pop dance; Taekwondo performance; validation

## Abstract

**Background:**

This study validates the Korean Movement Culture Perception (KMCP) scale, a tool designed to assess Korea's 'Movement Culture' (e.g., K-pop dance, Taekwondo), a key element of the 'Shin Hallyu' (New Korean Wave) characterized by participatory consumption. Using the Rasch model, this study analysed the validity of the scale among 1,240 participants from Korea and China, a major market for Hallyu.

**Methods:**

Participants responded to the 21-item KMCP scale, which comprises three sub-factors ('Korean attractiveness,' 'correct knowledge information,' and 'content diversification'), using a five-point Likert scale.

**Results:**

The scale demonstrated high internal consistency (Cronbach’s α = .954), and all 21 items showed an adequate model fit (Infit range: 0.86–1.11), confirming its overall construct validity. However, the item-person map revealed that item difficulty was clustered in the middle, indicating a limited capacity to measure diverse perception levels. Furthermore, significant differential item functioning (DIF) was found in five items (4, 6, 7, 12, and 31) based on nationality, with three of this exhibiting cultural bias unfavourable to foreigners.

**Conclusions:**

In conclusion, while the KMCP scale is a generally valid tool, the revision of culturally biased items is urgently needed to ensure cross-cultural equivalence. By improving the scale, this study will contribute to a more objective evaluation of Hallyu content's value and support the global dissemination of Korean movement culture as a health promotion tool through education.

## Introduction

Cultural strategy plays a crucial role in enhancing a nation’s identity and brand value in today’s global environment. ^1^ As globalization offers diverse choices to global consumers, cultural strategy directly influences consumer attitudes and purchase intentions. ^2^ Accordingly, many countries are formulating cultural strategies based on their cultural assets to secure global brand competitiveness. Korea has actively pursued a global cultural content development strategy centred around the Korean Wave (Hallyu) as part of its national cultural strategy. Hallyu refers to the global popularity of Korean popular culture, which initially expanded through K-dramas and K-pop, and has since evolved through diversified content, expanded platforms, and changing consumer demands. This evolution has given rise to the concept of “Shin Hallyu” (New Korean Wave). ^3^ Unlike earlier stages that focused on passive consumption, Shin Hallyu is characterized by “participatory cultural consumption,” in which global fans actively engage in experiencing and practicing Korean culture. This includes movement-based cultural content such as traditional Korean dance, Taekwondo, K-pop dance, Nanta (nonverbal percussion performance), and B-boying. In this study, the term “Movement Culture” is specifically defined as Korea’s embodied performance-based cultural practices, such as K-pop dance, traditional Korean dance, Taekwondo demonstrations, and Nanta (percussion performance). Unlike the general usage of “movement culture” in physical education or sport studies, here it refers to the creation of cultural value through embodied, nonverbal performance, which functions as both cultural content and a form of international cultural diplomacy within the Korean Wave (Hallyu). These practices constitute Korea’s unique form of “movement culture,” where cultural value is created through nonverbal, embodied performance. The emergence of such physical activity-based cultural content highlights the strategic potential of movement culture as a new form of cultural diplomacy.

According to a global Hallyu survey conducted by the Korea Foundation for International Cultural Exchange, the export value of Hallyu-related cultural products, including consumer goods and tourism, reached USD 9.48 billion, with an induced production effect of KRW 19.8 trillion and an added value effect of KRW 7.8 trillion. ^4^ These figures underscore the critical role of Hallyu content in enhancing Korea’s economy and national image. ^5^ While the success of Hallyu has attracted global attention, cultural scholars have debated the driving mechanisms behind it. ^6^ Early studies explained Hallyu through the lens of geographical proximity, arguing that Korea's cultural content was more readily embraced in neighbouring East Asian markets. ^7, 8^ However, this explanation falls short in accounting for Hallyu’s success in more distant markets such as North and South America and Europe. Consequently, researchers have shifted their focus to cultural strategy and the concept of cultural hybridity—the interplay between globalization and localization—as key factors behind Hallyu’s global expansion. ^9^ This hybridity is especially relevant in the context of Shin Hallyu, exemplified by “K-Heritage,” which fuses traditional Korean elements with global aesthetics. During the COVID-19 pandemic, traditional Korean cultural assets such as hanbok (traditional dress), hansik (Korean cuisine), traditional dance, and palace architecture were introduced to global audiences via K-pop music videos, lyrics, and drama settings, effectively serving as cultural mediators. ^10^ These efforts have contributed to the continued expansion of Hallyu’s influence, evidenced by the rise in foreign tourists seeking traditional cultural experiences, sold-out overseas performances of Korean traditional dance, and the popularity of online K-pop dance classes. ^11, 12^ Movement culture, defined as the creation of cultural meaning through embodied, nonverbal performance, encompasses a wide range of activities including traditional dance, Taekwondo performances, K-pop dance, Nanta, and B-boying. Such activities not only express cultural identity but also promote emotional and psychological resilience. Movement culture thus provides sustainable content that transcends age and background, contributing to Hallyu’s organic growth through cultural education. The World Health Organization (WHO) has emphasized the significance of combining art and physical activity, reporting that such integration positively impacts mental health. ^13^ From this perspective, Korea’s movement culture can serve as a platform for transmitting positive messages across the globe. Despite its success, Hallyu has faced challenges such as content homogenization, over-commercialization, national conflicts (e.g., anti-Korean sentiment and the Chinese THAAD-related restrictions), and limited opportunities for direct experience. ^14, 15^ In response, scholars have emphasized the need to better understand the mechanisms behind Hallyu and to move away from indiscriminate supply in favor of data-driven analysis of consumer perceptions and needs. For this purpose, the development of a valid and reliable evaluation tool is essential. Accordingly, Korean researchers have developed the Korean Movement Culture Perception (KMCP) scale to evaluate the perceived value of movement culture within Hallyu. In accordance with established psychometric development protocols, validation must first be ensured within the originating culture. ^16, 17^

Given that movement culture entails rich cultural interpretation, it is imperative to structure the tool based on the experiences and perceptions of Korean participants. Once the scale is validated domestically, it may be applied cross-culturally. This study seeks to validate the KMCP scale using the Rasch model, a one-parameter Item Response Theory (IRT) model, for both Korean and Chinese populations. China was selected due to its significance as the initial platform and one of the largest consumer markets for Hallyu, as well as the importance of understanding neighboring countries’ perceptions. Moreover, in the 2022 Global Hallyu Survey, Chinese respondents showed the highest willingness to pay for Korean cultural content. ^18^ Therefore, evaluating the perception of Korean movement culture—including among Chinese audiences—can provide a meaningful indicator for the sustainable development and dissemination of Hallyu. This study empirically verifies the construct validity of the KMCP scale and aims to present movement culture as a therapeutic, policy-relevant, and educationally valuable component of Shin Hallyu that can deliver positive messages globally.

IRT is divided into three models based on the number of parameters included in the model: 1-parameter Rasch model, 2-parameter logistic model, and 3-parameter logistic model. The Rasch model corresponds to the 1-parameter logistic model and was developed by the Danish mathematician Georg Rasch in the 1960s. It later became widely known through the work of Professor Wright in the Department of Education at the University of Chicago. ^19^ The Rasch model is a single-item response theory model that allows responses to specific items to be independently estimated using the same metric for individual abilities and item difficulties. ^20^ It is recognized for its methodological advantages in addressing problems not solved by classical test theory, including the ability to identify items that function differentially based on group characteristics and the invariance of parameter estimates based on group abilities or characteristics. ^21, 22^ Therefore, this study seeks to validate the KMCP evaluation tool, which assesses the value of Movement Culture, using the Rasch model to provide an objective measure and valid testing tool for the ongoing development of Hallyu, particularly in the context of promoting physical and cultural activities.

1. Does the KMCP scale demonstrate structural validity through Rasch model analysis across Korean and Chinese participant groups?
2. Do the items of the KMCP scale exhibit an appropriate distribution of difficulty levels to adequately measure participants across diverse ability ranges?
3. Does the KMCP scale function consistently without differential item bias across demographic characteristics such as gender, age, and nationality?
4. Can KMCP scores validly reflect participants’ positive perceptions and acceptance of Korean movement culture?

## Hypothesis and Methods

### Hypothesis

The hypotheses of this study are as follows. First, the study is designed to rigorously assess the construct validity of the Korean Movement Culture Perception (KMCP) scale within the context of foreign participants. Methodologically, the Rasch model is employed to conduct a comprehensive analysis. Furthermore, the research aims to delineate the nuanced impact of gender, age, and nationality on the multifaceted landscape of response validity. Second, the participant cohort, meticulously sampled to represent a diverse spectrum of age groups and national origins, is entrusted with a discerning evaluation of perceptual nuances encapsulated within the Korean exercise culture paradigm, facilitated through the KMCP scale. A theoretically grounded proposition posits that increases in KMCP scores inherently correspond to an elevated propensity toward embracing Korean movement culture. Third, in adherence to best practices within psychometrics, the forthcoming analytical examination of the KMCP scale data, founded on the Rasch rating scale model, is poised to consistently demonstrate the tenets of measurement validity across all facets of analysis. This further underscores the significance of the scale within the overarching research framework.

### Participants

To achieve the objective of this study, a sample group was selected from among individuals who have experienced the Movement Culture of the Korean cultural wave. Convenience sampling, a non-probability sampling method, was used. Before the survey began, all participants were fully informed about the purpose and procedures of the study, that the collected information would be used for research purposes only while ensuring anonymity, and of their right to withdraw from the study at any time. After confirming their understanding and voluntary agreement to participate, written informed consent was obtained from all participants.

The participants consisted of 1,240 individuals (44.6% male, 55.4% female) who were categorized into two age groups: 50% were in their teens and 20s, while the remaining were in their 30s to 60s. The rationale for categorizing age into “10s to 20s” and “30s to 60s” is that the classification reflects a nearly even distribution of data, with approximately 50% representation in each age group. The nationalities of the participants were evenly split between Korean and Chinese. Participants (approximately 87%) mainly comprise students (24%), office workers (33%), or professionals (individuals with knowledge and skills in specific fields such as medicine, law, pharmacy, etc.) (30%). In terms of marital status, unmarried participants (51.9%) slightly outnumbered married ones (48%). Of the total data, 39 responses were from minors aged 15–18, accounting for 3% of the total. The survey was conducted after obtaining parental consent from all minors. Table 1 presents the demographic characteristics of all participants.

**Table 1.** Demographic Characteristics (n=1240).

Characteristics		Frequency (%)
Country	Korean	624 (50.3)
	Non-Korean (Chinese)	616 (49.7)
	Total	1240 (100.0)
Gender	Male	553 (44.6)
	Female	687 (55.4)
	Total	1240 (100.0)
Age	10s–20s	618 (49.8)
	30s–60s	622 (50.2)
	Total	1240 (100.0)
Relationship status	Single	643 (51.9)
	Married	597 (48.1)
	Total	1240 (100.0)
Occupation	Student	296 (23.9)
	Office job	404 (32.6)
	Profession	376 (30.3)
	Homemaker	68 (5.5)
	Jobless	63 (5.1)
	Other	33 (2.7)
	Total	1240 (100.0)

### Korea Movement Culture’s Perception Scale and Procedure

The KMCP scale was developed specifically to measure the perception toward the Korean Movement Culture. ^20^ It consists of 21 items under three subcategories: (1) Korean attractiveness, (2) correct knowledge information, and (3) content diversification. Responses to the items are measured on a Likert 5-point scale (1 = Strongly disagree, 2 = Disagree, 3 = Neutral, 4 = Agree, 5 = Strongly agree). A higher KMCP response score can be interpreted as a more positive perception of the Korean Movement Culture. Prior to conducting the survey, the KMCP scale was translated into Chinese while maintaining its original consistency without adding new expressions or items. First, a professor (of Chinese nationality) from abroad, who is an expert in a KMCP-related field and fluent in Korean, conducted the first translation. Following the first round of translation, two researchers who had developed the KMCP scale and two KMCP-related field experts reviewed its clarity. Finally, five KMCP-related field and translation experts reviewed and modified the items until a consensus was reached regarding the expressions or sentences that were difficult to interpret. Data that corresponded to intentionally random responses or data with missing responses were to be excluded, but as none were found, all data collected were used. A pre- study conducted on the general Korean population showed acceptable reliability (Korean attractiveness Cronbach’s α =.914, correct knowledge information α = .893, and content diversification α = .836) and validity for the KMCP scale. ^19^

### Data Analysis

In this study, the Rasch rating scale model, a type of Rasch model ^21^ developed to analyze rating scale data, was used to analyze the KMCP scale data. The Rasch rating scale model uses the estimated item and person parameters to perform the analysis. Before examining the validity of the KMCP scale, a principal component analysis (PCA) was conducted to confirm whether the 21 items violated the basic assumption of unidimensionality in the Rasch model. Additionally, Cronbach’s alpha analysis was performed on all the 21 items of the KMCP scale. The results showed that the percentage of explained variance by the main factor was 57.8%, which satisfied the unidimensionality assumption required for analysis using the Rasch model. ^23, 24^

Cronbach’s alpha analysis also showed a high reliability coefficient of 0.954. The Winsteps (ver 3.65) program was used to conduct model data fit, rating scale functioning, item-person map, item and individual fit, and differential item functioning (DIF) analyses of the Rasch rating scale model. Based on the results of this study, the analysis method can be summarized as follows. Model data fit was evaluated using item fit indices (infit and outfit) for the KMCP scale items. Item fit indices were evaluated as appropriate when they were close to 1.0, with values below 0.5 indicating overfitting and values above 1.5 indicating poor fit. ^25– 28^

Rating scale functioning was evaluated using step calibration values and probability curves to assess the appropriateness of the response categories. The response categories were applied using a 5-point scale, and appropriateness was evaluated based on the following criteria: (a) an observed count of at least 10 responses for each category, (b) outfit values of less than 2.0 for each category, and (c) category thresholds that increased sequentially according to the intended scale and ranged from 1.4 to 5.0. ^29, 30^ The item-person map is a graph that visually displays the estimated distribution of subject’s KMCP scores and the estimated difficulty of the items on the same scale for easy comparison. The appropriateness of the scale evaluation was determined by assessing whether the two distributions of the subject’s KMCP score estimate and the estimated item difficulty are close to each other, and whether no items or subjects that are too high or too low on the graph existed. ^31, 32^

The study also evaluated the suitability of questions by using two statistical indices; infit and outfit and considered questions with either index over 1.50 or below 0.5 to be unsuitable. ^33, 34^ The higher the logit value of an item, the more difficult it was found to agree with. The item reliability index and the item separation index were calculated to verify the consistency of the item ranking by the participant group. These indices were deemed appropriate when the item reliability was 0.8 or higher and the item separation index was 2.0 or higher. ^35^ Individual ability estimates were presented using logit values, with higher values indicating higher ability. The person reliability index and person separation index were calculated to verify the consistency of the measurement and the stability of the ranking of participants based on the scale. The reliability and separation indices were set according to the same criteria as the item difficulty. Finally, the study performed a differential item functioning (DIF) analysis to determine whether any questions functioned differently for different groups based on gender, nationality, or age. The M-H DIF size was used to determine whether a question was discriminatory, with a value of 0.64 or higher indicating discrimination. A significance level of 0.001 was used to account for potential inflation across multiple comparisons. ^27, 36^

## Results

### Model Data Fit

The fit of the model data for the KMCP items was evaluated using the infit and outfit values. All 21 items showed appropriate statistical results. The infit range was 0.86–1.11, and the outfit range was 0.92–1.13 Table 3, which indicates that both ranges were within the acceptable range of 0.5–1.5. These results suggest that the 21 KMCP items were appropriate for measuring the primarily one-dimensional construct of the scale.

### Rating Scale Functioning

**Figure 1.**
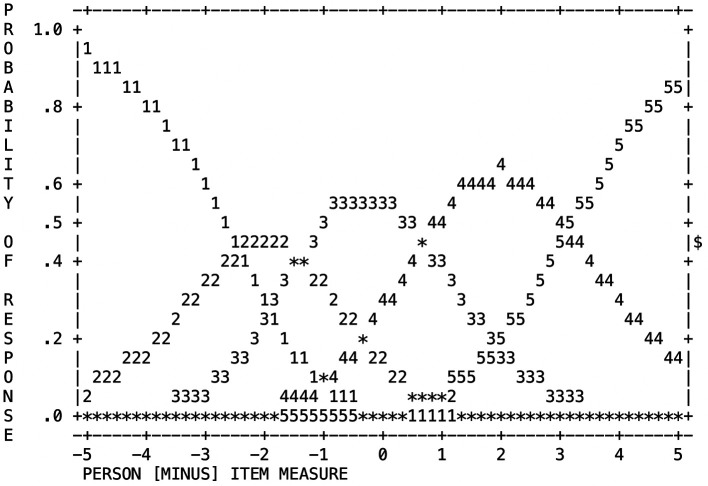
Five-point Likert Response Category Probabilities for the KMCP Scale.

Table 2 and Figure 1 present the results of the step calibration values and probability curves analysis for the 5-point KMCP scale to evaluate its fit. According to the results shown in Table 2, the distribution of counts used was uniform and regular. The scale improved as the number of categories increased, as indicated by the increasing average measure value. Outfit ranged from 0.97 to 1.58, or within the acceptable range of 2.0. Finally, category thresholds were found to increase sequentially according to the category scale, and the response categories were confirmed to have been selected a minimum of 50 times (4.2%) for 1 (not at all) and a maximum of 522 times (43.4%) for 4 (very much). These results indicated that the 5-point scale was appropriate for use with the KMCP scale.

**Table 2.** Summary of the KMCP Scale Rating Scale Function.

Category score	Counts used (%)	Average measure	Outfit MNSQ	Category thresholds
1	50(4.2)	-2.18	1.24	None
2	88(7.3)	-0.17	1.58	-2.42
3	315(26.2)	0.90	1.10	-1.41
4	522(43.4)	2.16	0.95	0.69
5	229(19.0)	3.12	0.97	3.14

*Note.* Average measure = mean of logit measures in category; MNSQ = mean square residuals.

### Item-Person Map

**Figure 2.**
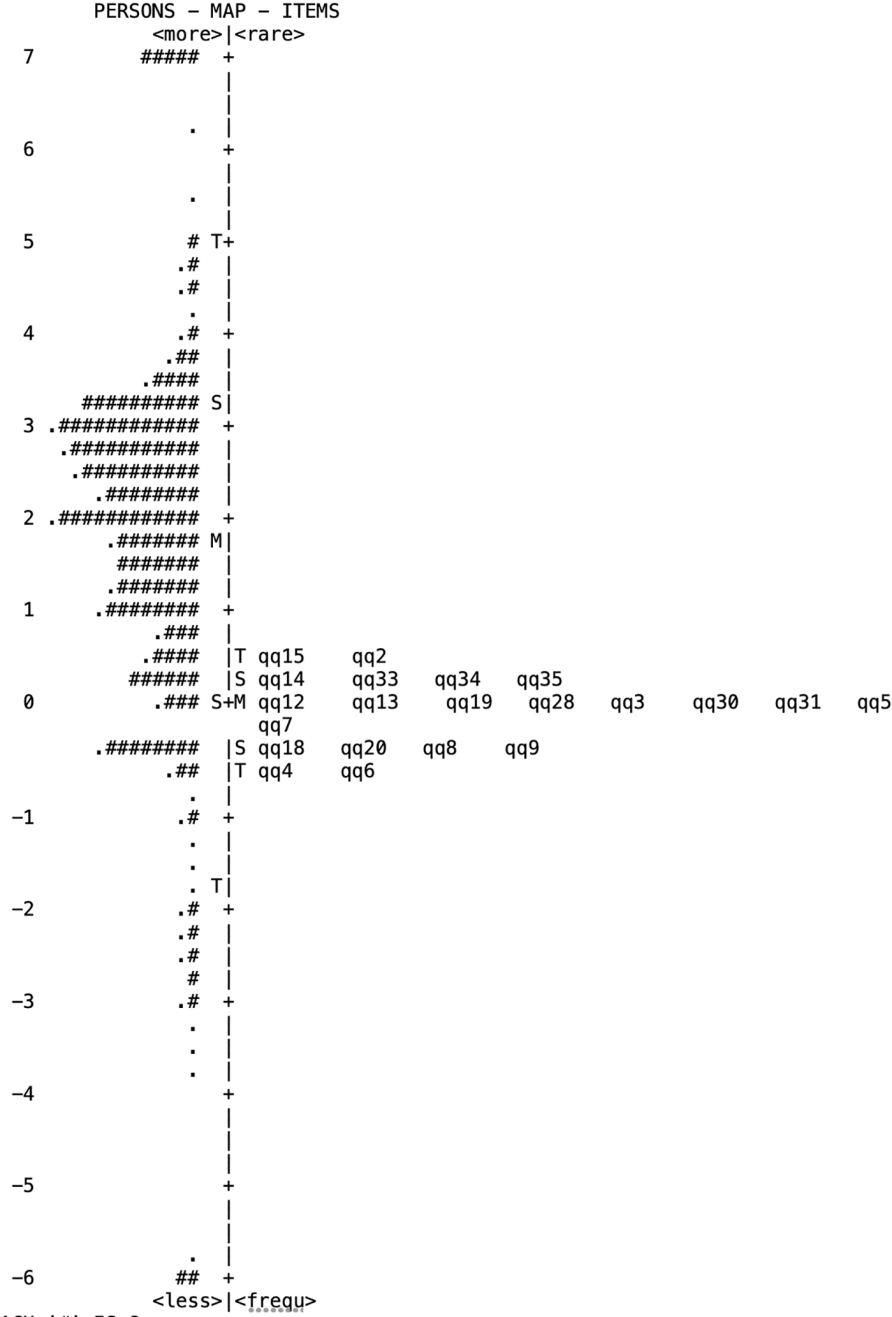
Item-person Map of the KMCP Scale. *Note*: Each “M” represents the mean of a participant’s level of KMCP on the left side and the mean of the corresponding item’s difficulty on the right side. “S” and “T” represent one standard-deviation and two standard-deviation points, respectively.

Figure 2 presents the item-person map of the KMCP scale, showing the estimated ability of the participants and the estimated difficulty of items on the same scale. On the left side of the map, the distribution of participant ability estimates is shown using the number sign (#) and dots, whereas on the right side, the distribution of item difficulty estimates is presented using item numbers ordered by difficulty. The numbers on the left represent logit values, where higher values indicate higher ability in relation to participants and higher difficulty for items. Based on the map, the distribution of item difficulty was relatively even; however, compared with the average participant logit, the difficulty of the items was concentrated in the middle. This suggests that, overall, the difficulty of the KMCP items is concentrated in the middle, and there is a shortage of items that can measure the range of participants’ KMCP levels, whether high or low. Therefore, item modification is necessary to ensure that the test can measure the full range of participants’ abilities.

### KMCP Item Difficulty

Table 3 presents the results of the analysis of item difficulty, standard error, Infit, and outfit in the KMCP scale. A higher logit value indicates a more difficult item that participants found harder to agree with. The range of item difficulty was -0.46 to 0.42. Specifically, the items that participants found the most difficult to agree with were, “Do you think there are many attractive performances or promotions?” and “Are there any convergence contents?” (logit = 0.42), respectively. The item that was the easiest to agree with was “Do you think Korea has its own unique charms?” (logit = -0.46). Looking at the reliability and separation indices of each item, the reliability index was.96 and the separation index was 4.79, with both meeting the acceptable criteria for reliability (.80 or higher) and separation (2.0 or higher).

**Table 3.** Item Summary of Rasch Calibration in the KMCP Scale. Item numbers refer to the original scale development phase.

Item	Calibration Logit	SE Logits	Infit MNSQ	Outfit MNSQ
2. Do you think attractive performances or promotions are conducted frequently?	0.42	0.05	1.07	1.11
3. Can you empathize with the excitement or spirit together?	-0.04	0.05	1.11	1.13
4. Do you feel Korea has a positive image?	-0.45	0.05	1.00	1.04
5. Would you like to visit Korea?	-0.12	0.05	0.94	1.01
6. Do you feel Korea has unique charms?	-0.46	0.05	1.04	1.10
7. Do you feel attracted to try using Korea movement culture content?	0.12	0.05	0.93	0.94
8. Does it stimulate your curiosity about Korea movement culture content?	-0.15	0.05	0.86	0.92
9. Do you want to introduce it to your friends or acquaintances?	-0.15	0.05	1.04	1.03
18. Does it stimulate your curiosity about Korea movement culture content?	-0.15	0.05	1.01	1.04
19. Do you feel there are many potential audiences?	-0.12	0.05	0.95	0.97
20. Do you think it can grow into representative Korea movement culture content?	-0.21	0.05	1.01	1.01
28. Do you help understand Korean culture?	0.08	0.05	1.02	1.05
30. Can you understand Korean emotions correctly?	0.00	0.05	0.92	0.97
31. Do you think there is storytelling or expression delivery present to help your understanding?	0.06	0.05	1.02	1.02
33. Do you provide knowledge about Korean culture?	0.17	0.05	0.94	0.94
34. Do you hold an interest in Korean history and traditions?	0.21	0.04	0.95	0.95
35. Do you provide correct information about Korean culture?	0.25	0.05	0.94	0.95
12. Do you think it is possible for this to be considered as educational content?	0.06	0.05	1.02	1.02
13. Do you think this content can be enjoyed with family?	-0.12	0.05	0.95	0.97
14. Does this contain a variety of contents such as tradition, modern, and art?	0.17	0.05	0.94	0.94
15. Are there fusion contents for each content category?	0.42	0.05	1.07	1.11

### Individual Level of KMCP

The KMCP levels of the study participants were estimated as logit values through a calibration process, where higher logit values indicate a higher level of KMCP. The average KMCP score of the participants was 1.67 (SD=1.67). The range of KMCP was between 6.18 and -5.69. When examining the reliability and separation indices of the participants, the reliability index was 0.94 and the separation index was 4.03, which meet the acceptable standards (reliability: .80 or higher; separation index: 2.0 or higher). These indicate that the individuals were well spread out along the continuum with a high degree of confidence in replicating person-placement within the measurement error.

### Differential Item Functioning

To confirm whether the KMCP scale functioned equally across different gender, nationality, and age demographics, a differential item functioning analysis was conducted. No items were found that functioned differentially across gender and age groups. However, for nationality, five items (4, 6, 7, 12, and 31) were statistically significantly different (p<0.001 and M-HDIF size >0.64 logit). Specifically, Items 4, 6, and 7 were more favourable for Korean participants (logits = -.88, -.82, and -.28) than for non-Korean ones (logits = -.04, -.10, and.53) in terms of strength of agreement (strongly agree or agree). Conversely, items 12 and 31 were more likely to be agreed upon by non-Korean participants (-.33, -.31) than by Korean ones (logits = .43, .41)

## Discussion

Based on the results of this study, the following conclusions were drawn. First, the statistical analysis of the KMCP test tool showed an adequate level of fit for all 21 items, indicating that it is suitable for measuring the one-dimensional construct that the KMCP scale aims to measure. Second, the analysis of the step-adjustment values and probability curves indicates that the 5-point scale used to assess KMCP is appropriate. Although the target population for the test tool has changed since its initial development, when the reliability and validity of the KMCP scale were established, the results of this study suggest that it maintains an adequate overall level of validity. Furthermore, the response frequency analysis shows that “Yes” was the most common response, accounting for 522 (43.4%) responses, and that most respondents had a positive attitude toward the KMCP items listed. A direct comparison with previous studies on the evaluation of Korean Wave content- related value indicators is difficult because of the lack of prior research with which to draw comparisons. However, one can infer that the positive responses were probably due to the significant impact of the Korea movement content and the factors that KMCP aims to measure, such as K-pop dance, Korean dance, taekwondo performance, b-boying, and Nanta. Third, upon examining the participants’ abilities (their awareness of the new Korea cultural contents) and the estimated difficulty levels of the questions, it was found that while the participants’ abilities were distributed across a range from high to low, the difficulty level of the questions was clustered around the middle, indicating a shortage of questions that could address all participants’ abilities. This result can be attributed to the fact that Korea movement content has spread not only in Asia, but also in a wide range of countries and regions including the United States and Europe. Since the abilities of most participants are distributed in the upper-middle range, the difficulty of the KMCP problem can increase if you ask a more profound question, such as “Does its express Korea’s unique sensibility well?” rather than simply asking “Is Korea attractive?” Looking at the research results of the cultural competency assessment (CCA) tool development study, ^37^ it was reported that the validity of cultural competency varied depending on the level of education. These results partially support the research finding that since the individual abilities of the participants in this study are diverse, the question format should be varied accordingly. As it has continued to develop globally, recognition and awareness of Korea Movement Culture, including not only K-pop dramas and entertainment but also K-pop dance, Korean traditional dance, Taekwondo performances, B-boying, and Nanta, has become very high. As a catalyst for global change, the Korean Wave has influenced global markets and transformed national relationships. ^38^ Fourth, a discrimination analysis was performed on the variables of gender, nationality, and age in relation to the KMCP scale, which revealed that five questions (questions 4, 6, 7, 12, and 31) had discriminatory functions for nationality. Questions 4, 6, and 7 asked about the positive image of Korea and the appeal of Korean content and tended to generate positive responses from Korean participants; this was thought to reflect their national or cultural pride. It is necessary to amend these questions to render them more objective, or alternatively to entirely remove them from the scale questionnaire. The 12th and 31st items were related to evaluating the New Korea movement content (Korean dance, Nanta, Taekwondo performance, K-pop dance, B-boying), and they elicited generally positive responses from foreign participants. Therefore, these items should be removed or revised so that people across nationalities can evaluate them objectively.

## Conclusions

Korean cultural content, also known as the Korean Wave, has become an essential element in the modern, hyper-connected global society. The purpose of this study was to verify the importance of Korean Movement Culture as part of the Korean Wave through evaluations by Koreans and foreigners (Chinese). This study confirmed the validity of objective evaluation indicators for the purpose of laying the foundation for the continued growth and development of the Korean Wave. The validity of the Korean Movement Culture Perception (KMCP) scale was investigated by applying a single-parameter Rasch model. The KMCP scale was developed for both Koreans and foreigners to determine whether the scale responded differently depending on demographic factors such as gender, age, and nationality. Through validity analysis, the validity of the KMCP scale was confirmed in all analyses. However, we also identified several differentiating items. As a result of the investigation, five items (4, 6, 7, 12, and 31) were found to function differently, and items 4, 6, and 7 were found to be unfavourable to foreigners. Therefore, by modifying the KMCP scale items based on these results, it will be possible to more accurately measure foreigners' perception of Korean Movement Culture. The purpose of this study is to promote the international and educational development of Korean Movement Culture, making it accessible to people of all genders and ages.

## Ethical approval and consent to participate

The data used in this study was collected without the collection of personal information and is not considered part of life science research. Based on information from the Korea National Institute for Bioethics Policy, which states that 'studies that do not include 'vulnerable subjects in a vulnerable environment' as research subjects and do not collect or record 'sensitive information' from research subjects, even if they use data obtained through interactions such as communication or face-to-face surveys of research subjects or observations of the behavior of research subjects, are exempt from review,' this research did not require ethical approval. However, prior to conducting the survey, I provided a clear and understandable explanation of the research's objectives, content, utilization, and safety precautions to all participants in this study. Furthermore, I obtained their voluntary consent through the signing of a pre-survey agreement, ensuring that they fully understood all aspects of the study before proceeding with the survey.

## Consent for publication

Not applicable.

## Data Availability

Data supporting the findings of this study are openly available in Figshare at the following DOI: https://doi.org/10.6084/m9.figshare.30498830. ^39^ Data are available under the terms of the Creative Commons Attribution 4.0 International license (CC-BY 4.0).
