# Supplementary material for: Validation of the Korean Movement Culture Perception (KMCP) Scale Using the Rasch Model: Evidence from Korean and Chinese Populations
Source: F1000Res. 2026 Jan 5;15:3. [Version 1] doi: 10.12688/f1000research.171305.1 (PMC13033135; doi:10.12688/f1000research.171305.1)
Supplement: Peer review history

# Reviewer report: Reviewer response for version 1

**DOI:** 10.5256/f1000research.188900.r468368

**Published:** 2026-03-28

**Authors:** Nurul Akqmie Badrul Hisham

**Affiliations:** 1 National University of Malaysia, Bangi, Selangor, Malaysia

First, I would like to commend the author on this study. It is good work, and I truly enjoyed reviewing it. The manuscript addresses a timely and significant topic. To further enhance the impact and clarity of the paper for an international journal, I offer the following suggestions for revision:

 1. Geopolitical and Terminological Clarity

 Geographic Specificity: For the benefit of a global audience, I recommend specifying ‘South Korea’ or ‘South Koreans’ throughout the text rather than using the broader term ‘Korea.’ Given that the international community understands North and South Korea as two distinct entities with different cultural and political contexts, this distinction is essential for academic precision.

 Consistency of Terms: The manuscript currently uses ‘Hallyu’ and ‘the Korean Wave’ interchangeably. I suggest defining both at the initial mention to assist readers unfamiliar with the subject and then selecting one term to use consistently thereafter. When paraphrasing existing literature, you may adapt the terminology to match your chosen style for better narrative flow; for direct quotes, brackets such as ‘[South] Korea’ can be used to maintain your paper's specific focus.

 2. Discussion and Accessibility

 Structural Flow: The Discussion section is currently quite dense, presented in a single, long paragraph. Breaking this section into smaller paragraphs would greatly improve readability and help the reader navigate your arguments more effectively.

 Interpretative Depth: I suggest providing a more detailed, descriptive explanation of the broad data analysis. The goal is to ensure the quantitative results are accessible to a wide academic audience. Elaborating on the arguments behind the findings will help bridge the gap between statistical findings and the broader cultural conversation.

 3. Literature Currency

 Recent Citations: To further emphasize a contemporary awareness of the topic, I recommend incorporating few additional journal articles published within the last 3 to 5 years. This will ensure the manuscript reflects the most up-to-date scholarly discourse in this rapidly evolving field.

Is the work clearly and accurately presented and does it cite the current literature?

Partly

If applicable, is the statistical analysis and its interpretation appropriate?

Yes

Are all the source data underlying the results available to ensure full reproducibility?

Yes

Is the study design appropriate and is the work technically sound?

Yes

Are the conclusions drawn adequately supported by the results?

Yes

Are sufficient details of methods and analysis provided to allow replication by others?

Yes

Reviewer Expertise:

mass communication; interculture communication; Korean wave studies; mediatization

I confirm that I have read this submission and believe that I have an appropriate level of expertise to confirm that it is of an acceptable scientific standard.

# Reviewer report: Reviewer response for version 1

**DOI:** 10.5256/f1000research.188900.r449855

**Published:** 2026-02-11

**Authors:** Renata Czekalska

**Affiliations:** 1 Jagiellonian University, Krakow, Poland

This study aims to validate the Korean Movement Culture Perception (KMCP) scale using the Rasch rating scale model in Korean and Chinese populations. The authors analyzed responses from 1,240 participants to a 21-item, five-point Likert scale measuring perceptions of Korean movement culture (e.g., K-pop dance, Taekwondo, traditional dance).

 Using Rasch modeling, the study examined item fit, rating scale functioning, reliability, item-person distribution, and differential item functioning (DIF) across gender, age, and nationality. The results indicated good overall model fit, high internal consistency, and appropriate rating scale performance. However, item difficulty was concentrated in the middle range, and five items showed nationality-based DIF. The authors conclude that the KMCP scale is generally valid but requires revision to improve cross-cultural equivalence.

 The manuscript is clearly structured and well organized, with clearly stated research objectives, theoretical background, and analytical approach. Key concepts and the Rasch model are adequately introduced, and the authors cite relevant psychometric and cultural literature. Increasing the use of recent international journal articles and reducing reliance on non-academic sources would further strengthen the literature base, although this does not undermine the scientific soundness of the study.

 The cross-sectional survey design combined with Rasch analysis is appropriate for scale validation, and the large, balanced sample supports the academic merit of the work. The study makes a valuable contribution by extending previous KMCP validation to a cross-cultural context and identifying biased items. The main limitation is the use of convenience sampling, which restricts generalizability. This limitation could be discussed more explicitly, and future research could consider probabilistic sampling methods, but the current design remains suitable for the study’s aims.

 The authors provide sufficient methodological detail regarding participants, scale development and translation, analytical procedures, fit criteria, and software used. This information is generally adequate to allow replication by researchers familiar with Rasch modeling. Minor gaps include the absence of the full questionnaire and limited description of survey administration procedures. Providing the complete instrument and clarifying data collection methods would improve transparency but are not essential for replication.

 The statistical analyses are appropriate and consistent with the study objectives. The authors correctly apply unidimensionality testing, reliability analysis, Rasch fit statistics, rating scale diagnostics, item-person mapping, and DIF analysis. The interpretation of results is accurate and aligned with Rasch theory, and the identification of item clustering and biased items is well supported by the data. 

 The authors make the dataset openly available through Figshare and specify licensing terms, which supports transparency and reproducibility. However, analysis scripts, syntax files, and detailed data documentation are not provided. Sharing these materials and including a data dictionary would further enhance reproducibility, although the availability of raw data already meets current standards.

 The conclusions are directly supported by the results. The authors appropriately state that the scale is generally valid, identify limitations related to item difficulty and cultural bias, and recommend revision rather than claiming full cross-cultural equivalence. Their interpretations are cautious and evidence-based. 

 Overall, the article meets accepted standards for psychometric validation research and is scientifically sound. The suggested revisions are minor.

Is the work clearly and accurately presented and does it cite the current literature?

Yes

If applicable, is the statistical analysis and its interpretation appropriate?

Yes

Are all the source data underlying the results available to ensure full reproducibility?

Yes

Is the study design appropriate and is the work technically sound?

Yes

Are the conclusions drawn adequately supported by the results?

Yes

Are sufficient details of methods and analysis provided to allow replication by others?

Yes

Reviewer Expertise:

Cultural Studies; Asian Studies; Intercultural Communication

I confirm that I have read this submission and believe that I have an appropriate level of expertise to confirm that it is of an acceptable scientific standard.
